# Underwater recordings of the whistles of bottlenose dolphins in Fremantle Inner Harbour, Western Australia

**DOI:** 10.1038/sdata.2017.126

**Published:** 2017-09-12

**Authors:** Sarah A. Marley, Christine Erbe, Chandra P. Salgado Kent

**Affiliations:** 1 Centre for Marine Science and Technology, Curtin University, Perth, Western Australia, Australia

**Keywords:** Marine biology, Biological physics, Animal behaviour

## Abstract

Dolphins use frequency-modulated whistles for a variety of social functions. Whistles vary in their characteristics according to context, such as activity state, group size, group composition, geographic location, and ambient noise levels. Therefore, comparison of whistle characteristics can be used to address numerous research questions regarding dolphin populations and behaviour. However, logistical and economic constraints on dolphin research have resulted in data collection biases, inconsistent analytical approaches, and knowledge gaps. This Data Descriptor presents an acoustic dataset of bottlenose dolphin (*Tursiops aduncus*) whistles recorded in the Fremantle Inner Harbour, Western Australia. Data were collected using an autonomous recorder and analysed using a range of acoustic measurements. Acoustic data review identified 336 whistles, which were subsequently measured for six key characteristics using Raven Pro software. Of these, 164 ‘high-quality’ whistles were manually measured to provide an additional five acoustic characteristics. Digital files of individual whistles and corresponding measurements make this dataset available to researchers to address future questions regarding variations within and between dolphin communities.

## Background & Summary

Animal communication sounds are diverse and dynamic. Delphinids produce a variety of sounds, which typically fall into three types: brief echolocation clicks, broad-band burst-pulse sounds, and frequency-modulated whistles. Whistles have received considerable research attention as a result of the role they play in social communication and because they can be easily characterised by their frequency contours. Functions of dolphin whistles include group cohesion^1^, coordination of group activities and movements^2,3^, identification of food patches^4^, and individual identification^5^. However, although our understanding of whistle function is developing, the reasons behind variations in these sounds remain poorly understood.

Dolphin whistles can be described using a number of acoustic characteristics that appear to vary with context, such as frequency components and duration. Whistle production is generally highest whilst dolphins are socialising^6^, with whistle characteristics also varying according to group activity state^7–11^. For instance, Heiler *et al.*^7^ found an upward shift in several frequency parameters during non-resting behaviours. Changes in acoustic behaviour can also occur in association with other factors, such as group size or composition^7,8,10,12,13^. For example, alterations in whistle characteristics have been correlated with the presence of calves in a group, with calf whistles reported to be significantly longer and lower in frequency than those of adults^14^.

Population differences in whistle characteristics also occur at the micro- and macro-geographic scale^6,15–18^. This variation could reflect geographic distance or genetic differences between populations of the same species^18^. Environmental conditions at different sites, such as levels of ambient noise, could also influence whistle characteristics. The underwater soundscape has spatio-temporal variations in its acoustic characteristics, as a result of abiotic, biotic and anthropogenic sound sources. Dolphins have been observed to alter their whistle characteristics in elevated noise conditions or in the presence of tourism boats^7,9,13,16,19,20^. Changes to whistle duration have also been reported^9,13,16^, as have increases in whistle production rates^13,21,22^. These changes are hypothesized to counter-act the ‘masking’ effect of anthropogenic noise.

In summary, dolphin whistles have high plasticity that allows dolphins to adapt to changes within the environment. Consequently, dolphin behavior can be better understood through analysis of their whistle characteristics. Thus, the whistles made available here can be used to this end.

**Figure 1:**
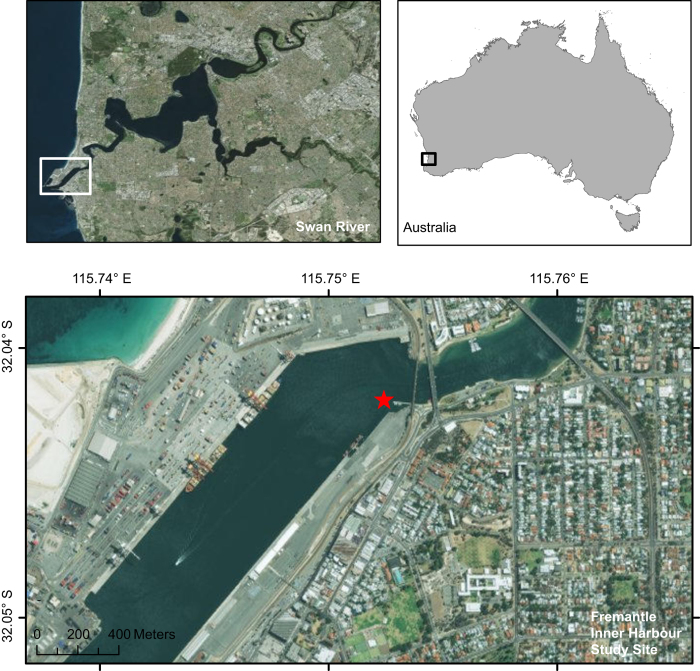
Map of the Fremantle Inner Harbour study area, showing the location of autonomous underwater acoustic recorder. Maps were created in ArcGIS (version 10.1) by Esri (www.esri.com) using the World Imagery basemap (sources: Esri, DigitalGlobe, Earthstar Geographics, CNES/Airbus DS, GeoEye, USDA FSA, USGS, Getmapping, Aerogrid, IGN, IGP, and the GIS User Community; http://goto.arcgisonline.com/maps/World_Imagery).

The whistles provided were collected during a study investigating the response of dolphins to underwater noise. The Swan-Canning Rivers in Perth, Western Australia, are part of an urban estuarine system that is home to a resident community of bottlenose dolphins (*Tursiops aduncus*). The underwater soundscape of the Swan River is composed of localized acoustic habitats which have high temporal variability, predominantly as a result of patterns in anthropogenic activities^23^. The anthropogenically-noisiest site in the Swan River is the Fremantle Inner Harbour (Fig. 1), which is the fourth largest cargo port in Australia and a gateway to the Indian Ocean from the Swan River for recreational and passenger ferry vessel traffic^23,24^. The harbour is also a foraging hotspot for the Swan River dolphin community^25,26^. Whilst dolphin whistles at this site have been described^17^ and animals have been observed to remain present in the harbor during periods of high vessel traffic^24^, the existence of acoustic responses to underwater noise had not been previously investigated. The data described here were therefore used to investigate variations in dolphin whistle characteristics in response to broadband noise levels and contextual variables such as group size, composition and activity state.

However, these data also have potential for re-use in studies conducting acoustic comparisons both within and between dolphin communities, as well as further behavioural analyses. Dolphin research has historically been, and continues to be, challenged by logistical and economic constraints. These constraints result in inconsistent analytical approaches, biases and knowledge gaps. For example, despite the flexibility of dolphin vocal production, many studies rely on only a small number of acoustic measures. Obtaining a broader picture of acoustical changes through measurement of a range of characteristics increases the power for detecting alterations in acoustic behavior. Additionally, as the majority of dolphin fieldwork is boat-based, there is a possibility of animals altering their acoustic behavior in the presence of research vessels. This is particularly problematic as it results in a lack of ‘control’ situations where no vessels are present. Therefore, remote collection of acoustic data is beneficial because it eliminates potential ‘observer effects’. By using an autonomous acoustic recorder and obtaining a range of whistle measurements in both the presence and absence of vessels, such as was done in this study, a dataset with reliable content can be re-used to answer further research questions.

## Methods

A high-frequency autonomous acoustic logger was deployed in the Fremantle Inner Harbour (32.0420S, 115.7528° E) from 30th April to 16th June 2015. The logger was assembled at the Centre for Marine Science and Technology (CMST); it was equipped with a programmable 16-bit digital sound recorder (made by Wildlife Acoustics Inc.) and an external hydrophone (Reson TC4033-1, sensitivity −202.8 dB re 1 μPaV^−1^) that entered the housing via a bulkhead connector to an impedance matching pre-amplifier with 20 dB gain. Digitised recordings were written to four 128 GB SD cards. Before deployment, the logger was calibrated by applying white noise of known power spectral density. An 8 Hz high-pass filter was employed to filter out high levels of low-frequency noise, thus enhancing the dynamic range of the recorder at the frequencies of interest. The logger recorded at a duty cycle of 10 min every 15 min at a sampling frequency of 96 kHz.

The logger collected approximately 1,080 h of acoustic data over the deployment period. This was beyond the capabilities of manual review so data were sub-sampled to periods which overlapped with land-based visual monitoring, which totalled approximately 35 h of concurrent visual and acoustic records. The acoustic data were manually reviewed in Adobe Audition (Vr 8.1.0.162) to identify the presence, number and location of whistles in each file. Whistles were visually graded based on their signal-to-noise (SNR) ratio (as per Heiler *et al.*^7^). Whistles were Grade 1 if the signal was faint, but visible on the spectrogram; Grade 2 if the signal was clear and unambiguous; and Grade 3 if the signal was prominent and dominant. Grades 2 and 3 were considered high-quality whistles, and only these were selected for further analysis. This ensured that only whistles with the entire contour clearly visible were measured, thus avoiding erroneous measurements due to masking of whistle components by other sources of noise.

**Figure 2:**
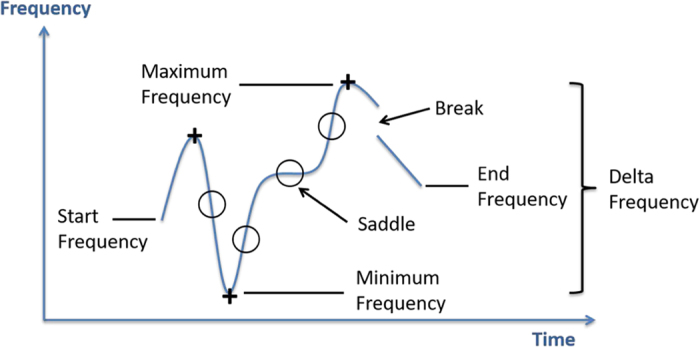
Example spectrogram showing several of the characteristics measured from each whistle. Inflection points are represented by ‘○’ and local extrema by ‘+’. Note that the saddle point is also an inflection point.

For all whistles, six acoustic characteristics were measured (Fig. 2): (1) *Duration*, (2) *Minimum Frequency*, (3) *Maximum Frequency*, (4) *Delta Frequency*, (5) *Start Frequency*, and (6) *End Frequency*. Characteristics 1–4 were automatically measured using the Raven Pro software (Vr 1.5), whilst characteristics 5 and 6 were manually measured from visual inspection. For the 164 high-quality whistles (Grades 2 and 3), an additional five acoustic characteristics were manually measured from visual inspection: (7) *Number of Inflection Points* (second derivative=0), (8) *Number of Extrema* (first derivative=0), (9) *Presence/Absence of Harmonics*, (10) *Number of Breaks*, and (11) *Number of Saddle Points* (multiple derivatives=0). Frequency measurements were rounded to the nearest 100 Hz.

We considered a whistle to be the curve of a continuous (in time) polynomial function and used established mathematical (calculus) definitions for the features of polynomials, including ‘*extrema’*, ‘*inflections’* and ‘*saddles’* (see below). As long as there was no break in time, a contour was considered one whistle. In other words, a whistle that was made up of a repetition of a shorter unit was still considered one whistle, so long as there was no temporal gap in the contour. Whistles occurring in trains with temporal gaps in between were considered separate whistles. The only deviation in the polynomial definition that we accounted for is an allowance for discontinuities in frequency, where the contour jumped (without any gap in time) from one frequency to another. We have called these discontinuities ‘*breaks’* (see Wang *et al.*^18^). This study does not consider the presence of signature whistles or multi-looped whistles^17,27^; however, some of the individual whistles reported here may in fact be loops from a disconnected multi-loop signature whistle.

Therefore, for the purposes of this paper (see Bronshtein and Semendyayev^28^), a local *extremum* of the whistle occurs at points where the first derivative (i.e., the slope) is 0 and the second derivative is negative (local maximum) or positive (local minimum). At a local extremum, the tangent to the curve is therefore horizontal (i.e., parallel to the time-axis). The slope is 0 and changes sign, either from positive to negative (local maximum) or from negative to positive (local minimum). An *inflection point* occurs at points where the second derivative (i.e., the curvature) is 0 and changes sign. The tangent to the curve intersects the curve at an inflection point. Sometimes, whistles plateau at local extrema or at inflection points. We called these plateaus ‘*saddle points’*. In calculus, they correspond to points on a curve where multiple derivatives are 0; and if the first non-zero derivative is of even order, then the point is a local extremum, if the first non-zero derivative is of odd order, then the point is an inflection point^28^. It is worth noting that some literature inter-changes these terms (e.g., an extremum here may be referred to as an ‘inflection’ elsewhere, or a saddle as a ‘step’).

Contextual information regarding the size and predominant activity state of dolphin groups was matched with high-quality whistles based on data from land-based visual surveys. Visual observations were collected by a team of 3–4 trained observers and recorded in real-time using Vadar (vr 2.00.01b; E. Kniest, University of Newcastle), as per previous studies at this site^24^. Visual data collection was limited to single 4 h surveys per day, conducted in good weather conditions (high visibility, Beaufort <3, low glare, temperatures <35C) to ensure accurate data and avoid observer fatigue. Observers scanned the area using 7×50 mm binoculars and a surveyor’s theodolite (TopCon GTS-603 AF Electronic Total Station) based at the top of a 32 m hill located approximately 300 m south of the Fremantle Inner Habour. If dolphins were sighted, an initial position was taken with the theodolite and the group size and activity state was noted. For these concurrent visual and acoustic records, only one dolphin group was present, with a ‘group’ being defined as ‘dolphins observed in apparent association, moving in the same direction and often, but not always, engaged in the same activity’^29^. Five activity states were used (Table 1): foraging, milling, resting, socializing, and travelling. These activity states were mutually exclusive and based on those used in other studies^30,31^. The predominant activity state was considered as the activity based on the most the most frequently observed behavior. A theodolite position was taken at every surfacing interval while dolphins were within the study area, with the exception of times when the surfacing interval was too brief to obtain an accurate reading. The dolphins were tracked until they left the study area, were lost from sight, or until conditions deteriorated sufficiently to justify ending the survey. To attribute contextual data, whistles were paired with a visual observation record based on the temporally closest theodolite data.

## Data Records

The acoustic recordings contained a total of 336 whistles when only one dolphin group was present in the study area, collected over 10 days from 230 min of acoustic recordings (Table 2). After visual assessment, 164 of these (captured over nine days from 220 min of acoustic recordings; Table 1) were considered sufficiently high in quality to be measured.

The dataset is available through an unrestricted repository at figshare (Data Citation 1). Each of the 336 whistles is stored as an individual.wav file, with a sequential name (Wh_0001, Wh_0002, Wh_0003, etc). File names correspond with a unique ‘Whistle Number’ in an Excel spreadsheet. The date and time of each 10 min acoustic recording are presented, along with a column called ‘Whistle Begins’ which lists the point within a recording when a whistle occurs. This spreadsheet also contains information regarding the whistle quality (Grades 1–3) and the whistle characteristics described above. Duration is measured in seconds, all frequency characteristics are measured in Hz, harmonics are present (1) or absent (0), and all remaining characteristics are counts.

A buffer of 0.2 s was included before and after each whistle. Consequently, the focal whistle always starts 0.2 s into the file. This allows the focal whistle to be easily identified, even if additional whistles occur in the file. Focal whistles can also be identified by their associated measurements in the Excel spreadsheet.

Four whistles do not have a 0.2 s buffer at the start of their respective files, as a result of the whistle occurring too close to the start of the recording. For example, if a whistle began 0.1 s into a recording, then it was not possible to provide a starting buffer 0.2 s in length. In these cases, the individual whistle file begins at the start of the acoustic recording. This applies to whistles Wh_0003, Wh_0004, Wh_0005 and Wh_0155.

## Technical Validation

The quality of the whistles recorded was assessed using a published methodology on dolphin whistle grading^7^. Several whistle characteristics were measured using Raven Pro software (Vr 1.5) (http://www.birds.cornell.edu/brp/raven). This software is an established and powerful tool for scientists working with acoustic signals, and has been used previously by the Centre for Marine Science and Technology (CMST) in order to measure dolphin whistles^17^. Manually collected measurements were agreed upon by two of the authors.

Summary statistics for whistle characteristics are presented in Table 3, including details on the minimum, maximum, mean and standard deviation values of the 164 high-quality whistles measured. This table excludes the characteristic harmonics, as this was a binary response variable. Harmonics were present in 35% (*n*=58) of high-quality whistles. Inflections were present in 88% (*n*=144), extrema in 49% (*n*=81), steps in 4% (*n*=6), and saddles in 12% (*n*=19) of high-quality whistles.

## Additional Information

**How to cite this article:** Marley, S. A. *et al.* Underwater recordings of the whistles of bottlenose dolphins in Fremantle Inner Harbour, Western Australia. *Sci. Data* 4:170126 doi: 10.1038/sdata.2017.126 (2017).

**Publisher’s note:** Springer Nature remains neutral with regard to jurisdictional claims in published maps and institutional affiliations.

## Supplementary Material



## Figures and Tables

**Table 1:** Definitions of dolphin activity states (adapted from Shane^29^ and Lusseau^31^).

**Activity State**	**Definition**
Foraging	Dolphins involved in any effort to capture and consume prey, often involving quick, steep dives of long duration. Diving birds or jumping fish may be observed.
Milling	Dolphins show frequent changes in heading, but stay in same location with no net movement.
Resting	Dolphins engage in slow movements or ‘logging’ at the surface.
Socialising	Dolphins engaged in a diverse number of interactive behavioural events, including body contact, chasing, leaping, or hitting the water surface with body parts. Groups may split or join.
Travelling	Dolphins engaged in persistent, directional movement with short, relatively constant dive intervals.

**Table 2:** Summary of acoustic data collected and dolphin whistles recorded.

**Date**	**Acoustic Files**	**Total Whistles**	**High-Quality Whistles**
8/05/2015	1	2	0
9/05/2015	3	59	49
15/05/2015	1	79	35
22/05/2015	2	38	14
29/05/2015	8	86	23
30/05/2015	1	1	1
8/06/2015	3	41	31
9/06/2015	2	11	4
11/06/2015	1	15	3
13/06/2015	1	4	4
Note that the acoustic recorder had a duty cycle of 10 min recording every 15 min, thus acoustic files were all 10 min in length.			

**Table 3:** Summary statistics the characteristics of a) all and b) high-quality whistles.

**a)**						
	**Duration (s)**	**Min Freq (kHz)**	**Max Freq (kHz)**	**Delta Freq (kHz)**	**Start Freq (kHz)**	**End Freq (kHz)**
Minimum	0.07	1.2	3.5	1.0	1.2	1.6
Mean	0.47 (±0.38)	5.0 (±1.9)	11.8 (±2.5)	6.8 (±3.1)	5.2 (±2.0)	11.3 (±3.1)
Maximum	4.86	11.9	17.8	14.2	12.6	17.8
Note that inflections, extrema, steps, and saddles were only recorded for high-quality whistles. Harmonics were not included as this was a binary response variable. Standard deviations are shown in brackets in the row with mean values.						

**b)**										
	**Duration (s)**	**Min Freq (kHz)**	**Max Freq (kHz)**	**Delta Freq (kHz)**	**Start Freq (kHz)**	**End Freq (kHz)**	**Inflections**	**Extrema**	**Steps**	**Saddles**
Minimum	0.07	1.2	3.7	1.0	1.2	2.1	0	0	0	0
Mean	0.59 (±0.48)	4.5 (±1.5)	11.8 (±2.5)	7.4 (±3.4)	4.7 (±1.7)	11.1 (±3.3)	2.7 (±2.4)	1.4 (±2.3)	0.1 (±0.7)	0.1 (±0.4)
Maximum	4.86	9.8	17.5	14.2	12.6	17.5	20	21	5	2
Note that inflections, extrema, steps, and saddles were only recorded for high-quality whistles. Harmonics were not included as this was a binary response variable. Standard deviations are shown in brackets in the row with mean values.										
